# Proteomic profiling of serum exosomes reveals acute phase response and promotion of inflammatory and platelet activation pathways in patients with heat stroke

**DOI:** 10.7717/peerj.16590

**Published:** 2023-12-13

**Authors:** Yue Li, Huan Li, Wenjuan Ma, Marc Maegele, Youqing Tang, Zhengtao Gu

**Affiliations:** 1 Academy of Orthopedics, Guangdong Province, Guangdong Provincial Key Laboratory of Bone and Joint Degenerative Diseases, Guangzhou, China; 2 Department of Treatment, Center for Traumatic Injuries, The Third Affiliated Hospital of Southern Medical University, Guangzhou, China; 3 Department of Intensive Care Unit, General Hospital of Southern Theatre Command of PLA, Guangzhou, China; 4 Department of Emergency Medicine, Guangdong Second Provincial General Hospital, Guangzhou, China; 5 Department of ICU, Sun Yat-sen University Cancer Center, Guangzhou, China; 6 Sate Key Laboratory of Oncology in South China, Sun Yat-sen University Cancer Center, Guangzhou, China; 7 University Witten/Herdecke (UW/H), Köln, German; 8 Department for Trauma and Orthopedic Surgery, Cologne-Merheim Medical Center (CMMC), University Witten/Herdecke (UW/H), Campus Cologne-Merheim, Ostmerheimerstr, Köln, Germany

**Keywords:** Heat stroke, Exosome, iTRAQ, Inflammation, Platelet activation

## Abstract

**Background:** The pathological mechanism of heat stroke (HS) involves the acute phase response, unbalanced immunological/inflammatory reactions, and coagulation initiation, especially platelet activation. Although exosomes contain proteins involved in these biological processes, their protein cargo levels and potential roles in HS remain unknown. This study explored the serum exosome protein expression patterns after HS and their potential roles in the pathogenesis of HS.

**Methods:** Blood samples were collected from ten patients diagnosed with HS upon admission to the intensive care unit (six with severe HS and four with mild HS). Samples from six healthy volunteers were included as control. Using ultracentrifugation, exosomes were prudently isolated, and their protein contents were profiled using liquid chromatography–tandem mass spectrometry analysis with isobaric tags for relative and absolute quantification-based proteomics.

**Results:** Compared with healthy volunteers, patients with HS showed significant changes in the levels of 33 exosomal proteins (23 upregulated and 10 downregulated). The most upregulated proteins included serum amyloid A-1 (SAA-1), von Willebrand factor (vWF), S100A8, and histone H3. In addition, SAA-1, vWF, platelet membrane glycoprotein, S100A8, and histone H3 were more enriched in the exosomes from patients with severe HS than from those with mild HS. Gene ontology analysis revealed that the HS-modulated exosomal proteins were mostly related to inflammatory response, including the acute-phase response, platelet activation/degranulation, and innate immune response. Kyoto Encyclopedia of Genes and Genomes pathway analysis revealed significant enrichment of proteins in the IL-17 signaling pathway, platelet activation, neutrophil extracellular trap formation, Fc epsilon RI signaling pathway, chemokine signaling pathway, and NOD-like receptor signaling pathway, among others. Several serum exosomal proteins, including SAA-1, vWF, and S100A8, which are related to the acute phase, inflammatory response, and platelet activation, were confirmed to be elevated in patients with HS, and were significantly correlated with disease severity, organ dysfunction, and death.

**Conclusion:** Overall, this study explores the potential role of the serum exosomal proteome in the inflammatory response and platelet activation in HS, suggests the pathological mechanisms underlying HS-induced injuries, and recommends reliable exosomal biomarkers for predicting HS prognosis.

## Introduction

Portions of this text were previously published as part of a preprint (Li et al., 2022; Xu et al., 2023).

A cause of mortality in military training or intense exercises, heat stroke (HS) is a great challenge to intensivists, owing to the unavailability of optimal evidence-based treatment. Lately, the incidence of HS has been on a rise due to frequent heat waves, with a high mortality rate of 40–60% (Epstein, Yanovich & Heatstroke, 2019; Hifumi et al., 2018).

The pathogenic mechanism of HS at the molecular level is complex and incompletely understood (Bouchama et al., 2005). HS may be induced by an imbalance in coagulation and inflammation cascades (Roberts et al., 2008). Recently, the roles of circulating extracellular vesicles, which are often associated with disease pathophysiology, have been extensively investigated (Reid & Webster, 2012). Hepatocyte-derived extracellular vesicles including exosomes can activate inflammatory signaling and the programmed cell death pathway in the liver, thereby leading to liver injury (Li et al., 2019, 2020). Additionally, exosomes with differentially expressed microRNAs (miRNAs) are associated with coagulation disorders and inflammatory reactions in patients diagnosed with HS (Li et al., 2021).

As exosomes exert multiple biological effects, RNA and protein cargo profiles can shed more light on the relationship between exosomes and disorders and help discover new diagnostic and prognostic markers. HS is considered as a “sepsis-like” syndrome, in which the systemic inflammatory response triggered by hyperthermia insult and resulting in end organ injury/dysfunction is very similar and comparable to conditions such as sepsis, severe multiple trauma, and burns. For example, the exosome proteomic profiling in septic cases is different from that in normal controls in terms of inflammation and the acute phase response (Real et al., 2018). Profiling exosomes in septic mice has revealed that cytokine/chemokine-enriched exosomes have important functions in the growth, differentiation, and chemotaxis of T cells in sepsis (Morris et al., 2020). Based on the proteomic analysis of sepsis-derived macrophages, exosomes demonstrate a downregulation of some significant protein networks, such as the immunomodulation process (Gao et al., 2019). In polytrauma patients, study on serum exosomal cargo can provide critical insights in the trauma-specific, temporal immune dysregulation, which may probably serve as unique biomarkers and therapeutic targets for timely and precise intervention (Walsh et al., 2021). In burn patients, Qin et al. (2020) found that 31 proteins were differentially expressed in serum exosomes. Of particular note in their findings was the up-regulation of ITGA2B and ITGB3 in the burn group, as these proteins are involved in the PI3K/AKT signaling pathway that promotes cell proliferation and growth (Qin et al., 2020). Furthermore, a relationship between abundant exosomal proteins and disease pathogenesis has been revealed and some candidate exosomal proteins have been identified as new biomarkers for organ damage (Walsh et al., 2021; Wisler et al., 2020).

Nonetheless, the protein profiles in exosomes during HS have not been analyzed so far. Therefore, this study focused on the analysis of alterations in serum exosomal proteomic profiles in HS, which may promote understanding of HS pathophysiology and provide possible research opportunities for developing novel disease-related biomarkers and treatments.

## Methods

### Patient screening and clinical data extraction

For the plasma exosome proteomic profile study, 10 patients, who were admitted to the ICU within the initial 24 h after HS from June 2019 to June 2021, were enrolled (four patients with mild HS and six with severe HS). The diagnosis of HS was made based on China’s Expert Consensus on Standardized Diagnosis and Treatment for Heat Stroke (2019), formulated by the Prevention and Treatment of Heat Stroke and Critical Care Committee of PLA of China (Qiu et al., 2021). Patients with active cancer, chronic renal or hepatic disorders, chronic pulmonary or cardiac insufficiency (New York Grades 3–4), concurrent central nervous system (CNS) diseases, or metabolic diseases or those who took antithrombotic medication such as heparin upto 3 weeks before the study began were excluded from the study. In addition, six healthy volunteers at the Physical Examination Center of this hospital were recruited to serve as the control group.

For the validation study (to verify the potential of targeted proteins in categorizing HS severity and predicting mortality), 50 HS patients were enrolled (in line with the same inclusion and exclusion criteria) from the ICU and categorized as those with mild HS (*n* = 29) or severe HS (*n* = 21). Thirty healthy individuals were enrolled from the Physical Examination Center as the control group. Basic demographic features and organ function information (plasma levels) of all subjects were extracted at admission (Day 1). Disease severity was assessed by age, APACHE II (chronic health evaluation), and sequential organ failure assessment (SOFA) scores. Blood samples were collected on Day 1. All patients or their family members gave informed consent, and the study was approved by the Medical Ethics Review Committee of the General Hospital of Southern Theater of the PLA of China (approval number: 20191808). Written informed consent from participants of our study was received.

### Blood sampling and exosome extraction

Peripheral venous blood sample (30 mL) was acquired from each enrolled individual. The initial 5 mL was discarded; the rest was added to ethylene diamine tetraacetic acid (EDTA) anticoagulant tubes and incubated at 22–27 °C (room temperature) for 30 min. Thereafter, the blood samples were centrifuged for 10 min at 2,500×*g* at 4 °C to extract the plasma. Protease inhibitors (containing 1 µg/mL pepstatin, 1 µg/mL aprotinin, and 3 mM phenylmethylsulfonyl fluoride) were added to the obtained plasma sample, which was then preserved at −80 °C for subsequent analysis. The plasma samples were diluted with an equal amount of phosphate-buffered saline (PBS) and subjected to a series of centrifugation steps at 4 °C (2,500×*g*, 12,000×*g*, and 110,000×*g* for 30, 45 min, and 2 h, respectively; SW 28 Ti Rotor, Optima L-90K Ultracentrifuge; Beckman Coulter, Fullerton, CA, USA). Finally, the precipitates (exosomes) were re-suspended with PBS (50–200 µL) and preserved at −80 °C.

### Exosome morphology observation by transmission electron microscopy (TEM)

Exosomal morphology was observed under TEM. Exosomes were first dehydrated with 2% formalin. Thereafter, an exosome suspension (5 mL) was added to a copper grid coated with formvar (Mecalab, Quebec City, Canada), which was incubated for 30 min at 4 °C. After rinsing with 100 µL of PBS, the sample was subjected to a 10 min fixation using 2% paraformaldehyde, as well as a 15 min staining using 2% uranyl acetate (supplemented within 50% ethanol). Afterward, samples were examined using the Philips CM10 transmission electron microscope (JEM-2100F).

### Nanoparticle tracking analysis (NTA)

Distributions of exosome levels and sizes were determined *via* NTA using NanoSight NS3000 (Malvern Instruments, Worcestershire, UK). Briefly, the exosome samples were diluted with sterile PBS at a ratio of 1:5,000, which was then divided into three equal parts and analyzed using NTA software (version 3.1) from NanoSight Ltd. (Amesbury, UK) and NanoSight LM10 for 60 s.

### Western blotting (WB) assay

Briefly, 1 mL of plasma was ultracentrifuged for 2.5 h at 120,000×*g* to extract the proteins with DE buffer (12 mM 2-mercaptoethanol, 20 mM Tris-HCL, 1 mM EGTA, 1 mM EDTA, 1% Triton-X 100, and 10% glycerol), which comprised a mixture of protease inhibitors (GE Healthcare, Uppsala, Sweden). Bradford assay was used to quantify total proteins. Then, aliquots of proteins (5 µg) of all samples were separated using 12% SDS-PAGE. Subsequently, the separated proteins were transferred onto nitrocellulose membranes, followed by overnight incubation at 4 °C with anti-CD9 (1:1,000, ab10895), anti-CD63 (1:1,000, ab92726), and anti-Tsg-101 (1:1,000, ab30871) (Abcam, Cambridge, UK) polyclonal antibodies as well as anti-GAPDH antibodies (endogenous reference). Densitometry was conducted to measure target protein level using ImageJ software.

### Comparison of relative protein profiles

#### Isobaric tags for relative and absolute quantification (iTRAQ)

During iTRAQ labeling, the peptide mixture (100 μg) was obtained in serum exosomes from both HS and control groups. Thereafter, labeling was performed following specific instructions (Applied Biosystems, Foster City, CA, USA). The protease was subsequently digested throughfilter-aided sample preparation (FASP). Proteins (150 ug) in every sample were reduced using 100 mM DTT under 100 °C for a 5-min period. Then, DTT, the detergent, and other low-molecular-weight components were removed using UA buffer (8 M Urea, 150 mM Tris-HCl pH 8.5) by repeated ultrafiltration (Sartorius, 30 kD). Afterwards, 100 μl iodoacetamide (100 mM IAA in UA buffer) was added to block the reduced cysteine residues and the samples were incubated for 30 min in dark. Later, the filters were washed with 100 μl UA buffer thrice and later with 100 μl of 50 mM NH_4_HCO_3_ buffer twice. Finally, the protein suspensions were digested with 4 μg trypsin (Promega) supplemented in 40 μl of 50 mM NH_4_HCO_3_ buffer overnight at 37 °C, and the resulting peptides were collected as a filtrate. Thereafter, the peptide segment was desalted with the C18 column. The peptide content was estimated based on the UV light spectral density at 280 nm using an extinction coefficient of 1.1 of 0.1% (g/l) solution calculated based on the frequency of tryptophan and tyrosine in vertebrate proteins.

### Peptide fractionation

The mixture of peptide segments from different samples was subjected to reduction, alkylation, and enzymatic cleavage in succession. Then, each peptide segment was labeled with different iTRAQ reagents (by using the 4plex reagent) including three parts, namely, reporter group (with relative molecular weights of 114, 115, 116, and 117 Da, respectively), balance group (with relative molecular weights of 31, 30, 29, and 28 Da, respectively), and peptide reactive group. The total molecular weight of reporter groups and balance groups in four iTRAQ reagents was maintained at 145 Da. No matter which iTRAQ reagent was used, the molecular weight of the same peptide segment labeled with different isotopes was identical in the primary mass spectrometry and presented in the same peak. The peptide reactive group connected the reporter group to the N-terminal and lysine side chains of the peptide, thereby labeling the reporter and balance groups on the peptide segment, and this contributed to labeling almost all proteins in the sample. In this way, all peptides in the same sample were labeled and all the labeled protein samples were mixed. Thereafter, tandem mass spectrometry was performed on the mixed labeled protein samples to obtain a primary mass spectrum. Samples from the same peak in the primary mass spectrometry were collected, and the same peptide end mixture of different samples was obtained. Subsequently, the protein sample was collected and secondary mass spectrometry was performed on the labeled peptide segments to obtain a secondary mass spectrometry image. Thereafter, the bonds between the reporter group, balance group and peptide reaction group were broken, and the balance group was lost. The same peptide with different isotope labels produced different reporter ions with molecular weights of 114, 115, 116 and 117 Da. The reporter ions presented different peaks. The height and area of the peaks and other related data were processed by software and compared with the database to obtain quantitative information of the same peptide segment among different samples. The peptides labeled with iTRAQ underwent fractionation using an AKTA Purifier system (GE Healthcare) *via* strong cation exchange (SCX) chromatography. After the reconstitution of the dry peptide mixture, buffer A (containing 10 mM KH_2_PO_4_ in 25% ACN with a pH of 3.0) was added to acidify the sample, which was loaded onto the PolySULFOETHYL column (5 µm, 4.6 mm × 100 mm, 200 Å) (PolyLC, Inc., Columbia, MD, USA). Thereafter, peptides were eluted at 22, 47–50, and 50–58 min by 0–8%, 8–52%, and 52–100% buffer B (10 mM KH_2_PO_4_ and 500 mM KCl in 25% ACN with a pH of 3.0), respectively, and reset using 0% buffer B at a flow rate of 1 mL/min. Subsequently, absorbance (OD) was measured at 214 nm to monitor the elution, and fractions were obtained at an interval of 1 min. After collection, diverse fractions were desalted with C18 Cartridges (volume 3 mL, bed I.D. 7 mm, Empore™ SPE Cartridges C18 at standard density; Sigma-Aldrich, St. Louis, MI, USA).

### High-performance liquid chromatography (HPLC)

The obtained fractions were loaded for nanoLC–MS/MS analysis (the operating temperature was 15–30 °C). Furthermore, the mixture of peptides was put on a reverse-phase trap column (Thermo Fisher Scientific, Waltham, MA, USA; nanoViper C18, and Acclaim PepMap100; 100 μm × 2 cm). The fractions were also subjected to a C18 reversed-phase analytical column (with a length of 10 cm, an inner diameter of 75 μm, and a resin of 3 μm; Thermo Fisher Scientific Easy Column; Thermo Fisher Scientific, Waltham, MA, USA) in buffer A (composed of 0.1% formic acid). Then, the resultant components were isolated using gradient elution with buffer B (with 84% acetonitrile and 0.1% formic acid) at a flow rate of 300 nL/min and regulated by applying IntelliFlow technology. The elution process lasted for 2 h using 0–55%, 55–100%, and 100% buffer B for 110, 5, and 5 min, respectively.

### LC–MS/MS analysis

The Q Exact mass spectrometer (Thermo Fisher Scientific, Waltham, MA, USA) operating in the positive-ion mode, which was equipped with Easy nLC (Proxeon Biosystems, formerly Thermo Fisher Scientific), was used to conduct LC–MS/MS analysis for 120 min. Additionally, the data-dependent method was also utilized to dynamically select abundant precursor ions from the survey scans (350–1,800 m/z), aiming to acquire MS data for higher-energy collisional dissociation (HCD) fragmentation. In addition, the maximal injection time (maxIT) and automatic gain control target were set at 50 ms and 3e6 separately. Survey scans with the resolution of 70,000 were acquired at m/z 200 using the maxIT of 50 ms and the AGC target of 3e6. Moreover, MS2 scans with the resolutions of 17,500 and 45,000 were acquired for HCD spectra at m/z 200 using the maxIT of 45 ms and the AGC target of 2e5, with the isolation width being set at 1.6 m/z. In this work, only ions whose charge state was 2–6 and the minimal intensity was 8e3 were selected in fragmentation. Moreover, the dynamic exclusion for those chosen ions, collision energy and standardized isolation width were 30 s, 30 ev and 2 m/z, respectively.

MASCOT engine (Matrix Science, London, UK; version 2.5) installed in Proteome Discoverer 2.1 was utilized for processing MS/MS raw files, which were also searched in corresponding database (Uniprot_HomoSapiens_20377_20220308_swissprot). The search parameters involved trypsin that served as an enzyme for generating peptides, and at most two missed cleavages were allowed. For MS2 fragments, their precursor mass tolerance was specified as 10 ppm and the tolerance was specified as 0.05 Da. Carbamidomethyl (C) was the constant modification, with the exception for iTRAQ labels. Variable modifications included Acetyl (Protein N-term) and Oxidation (M). Moreover, the reverse database search method was adopted for enforcing the peptide and protein false discovery rate to 1%. Phosphopeptides whose fold change >1.2 and *p*-value < 0.05 were shown to be potential differentially expressed proteins. Additionally, a peptide segment containing at least six amino acids was matched with a protein sequence database to identify the protein source. Universal Protein Resource (UniProt) was used as the standard protein identifier.

The mass spectrometry proteomics data have been deposited to the ProteomeXchange Consortium (http://proteomecentral.proteomexchange.org) *via* the iProX partner repository with the dataset identifier PXD040640.

### Gene ontology (GO) and Kyoto Encyclopedia of Genes and Genomes (KEGG) pathway enrichment analysis

Blast2GO was adopted for the GO analysis of target protein clusters (Proctor et al., 2020) in the following four processes: alignment of sequences (Blast), extraction of GO terms (Mapping), GO functional annotation (Annotation), and supplementary annotation (Annotation Augmentation). NCBI BLAST+ sequence alignment approach (ncbi-blast-2.2.28+-win32.exe) was applied to facilitate target clusters of proteins to align with suitable databases of protein sequences. The 10 most important alignment sequences (E-values ≤ 1e–3) were chosen for performing subsequent analyses. Later, GO protein terms related to the resultant suitable alignment sequences and target protein clusters were extracted using Blast2GO Command Line database (go_201504.obo; website: www.geneontology.org). The extraction allowed for the annotation of GO terms obtained during mapping into target protein sequences. The GO terms were annotated by considering the similarities between alignment and target protein sequences, GO term source reliability, and GO-directed acyclic graph structure. For the improvement of the annotation effect, we searched conserved motifs, matched them with target protein based on the EBI database by adopting InterProScan (Hifumi et al., 2018), and annotated the functional data associated with the target protein sequence motif. ANNEX annotation was also conducted, and annotation accuracy was improved by establishing connections among diverse GO categories.

Within the KEGG database, KEGG Orthology (KO) is a system applied to sort genes and their corresponding products. Among them, orthologous genes and their corresponding products, which had close functions within the identical pathway, were clustered and given the identical KO (or K) tag. While using target protein clusters to annotate KEGG pathways, we categorized target protein sequences into KO groups after comparing them with the KEGG GENES database with the application of the KEGG Automatic Annotation Server (Geng et al., 2015).

During the GO or KEGG analysis of target protein clusters, we compared GO or KEGG pathway distribution within target and overall protein clusters using Fisher’s exact test. Subsequently, we evaluated significant protein enrichment for GO terms or KEGG pathways.

### Assay for measuring plasma amyloid A-1 (SAA-1), von Willebrand factor (vWF), and S100A8 levels

SAA-1, vWF, and S100A8 levels within the plasma exosomes were detected using ELISA kits (Human Histone H3 ELISA Kit; Meimian Industrial Co., Ltd., Jiangsu, China) in accordance with the manufacturer’s protocols.

### Statistical analysis

Data distribution was checked using the kurtosis test. Normally distributed data are displayed as means ± standard deviation (SD), and abnormally distributed data as medians ± interquartile ranges (IQR). We also conducted a Student’s *t*-test (two-tailed) to compare normally distributed data between groups. Furthermore, categorical data were analyzed using Fisher’s exact test, whereas abnormally distributed data were analyzed using the Kruskal–Wallis H test. Multiple groups were compared using one-way ANOVA in combination with between-subjects factors, which was followed by the *post-hoc* Bonferroni correction for simple main effects. Categorical data were compared using McNemar’s test. Moreover, Spearman’s rank correlation test was conducted for the correlation analysis. Receiver operating characteristic (ROC) curves were plotted to assess the expression capacities of target exosomal proteins to discriminate non-survivors from survivors. Blast 2 and KASS software were used for GO annotation and KEGG pathway enrichment, respectively, using Fisher’s exact test. For the simultaneous classification of protein and sample modulation dimensions, we conducted a cluster analysis using Cluster 3.0 software. Java TreeView software was adopted for generating a hierarchical clustering heat map. All tests were performed using GraphPad version 7.0 (San Diego, CA, USA). Statistical significance was set at *P* < 0.05.

## Results

### Plasma exosome characterization

**Figure 1:**
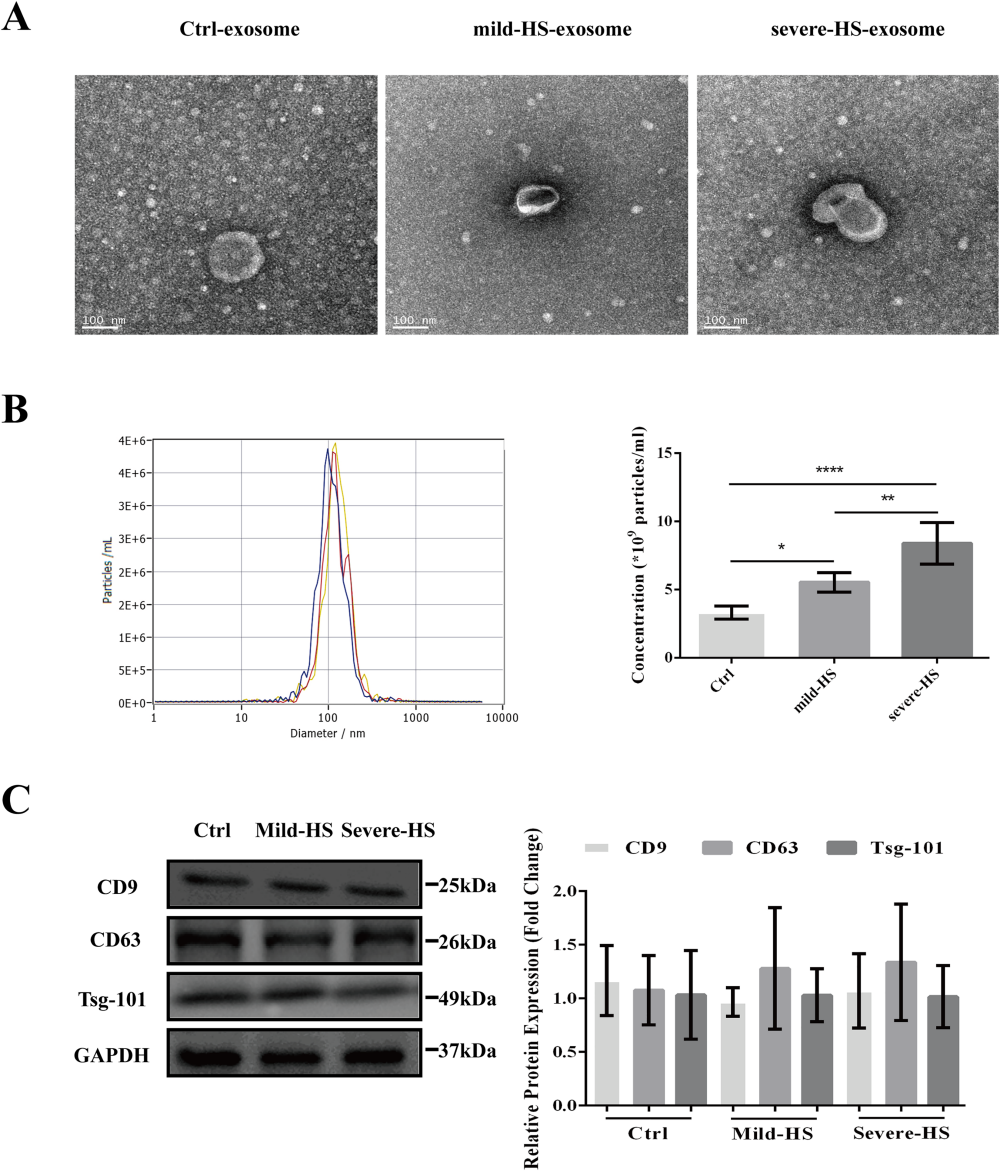
Representation of the plasma exosomes in healthy controls (Ctrl) and patients with mild heat stroke (HS) and severe HS from the aspects of surface markers, morphology, and diameter. (A) Morphology of visualized plasma exosomes with transmission electron microscopy (bar = 100 nm). (B) Size distributions and concentration of the exosomes from the Ctrl, mild HS, and severe HS groups obtained through nanoparticle tracking analysis (NTA). Left panel: the sizes of exosomes were between 30 nm and 150 nm (115.0 ± 47.4 nm for Ctrl, blue curve; 129.6 ± 54.9 nm for mild-HS, yellow curve; and 132.3 ± 49.8 nm for severe-HS, green curve). Right panel: the concentrations of plasma exosome in Ctrl and mild- and severe-HS groups. ****P < 0.0001, **P < 0.01, *P < 0.05 were used in the *post-hoc* test and one-way ANOVA. (C) Western blotting of mild HS, severe HS, and Ctrl groups’ plasma exosome proteins marked with typical exosomal markers (Tsg-101, CD9, and CD63). The left panel displays the bands that are representative and the right panel exhibits the relative levels of the proteins compared with those of GAPDH. Each test was repeated three times. All data are in the form of mean ± SEM.

As revealed by TEM, the serum exosomes isolated from control subjects as well as from cases with mild and severe HS showed double membranes and were vesicle-like structures with diameters of approximately 100 nm (Fig. 1A). In addition, NTA revealed that the plasma exosomes had comparable diameters among the three groups (control group: 115.0 ± 47.4 nm, mild-HS group: 129.6 ± 54.9 nm, and severe HS group: 132.3 ± 49.8 nm, respectively). Exosomal concentrations in groups with mild (5.53 ± 0.70 × 10^9^ particles/m) and severe HS (8.38 ± 1.53 × 10^9^ particles/mL) were higher than those in the control group (3.32 ± 0.47 × 10^9^ particles/mL) (*P* < 0.05). Moreover, their concentration in the group with severe HS was higher than that in the group with mild HS (*P* < 0.05) (Fig. 1B). Finally, the levels of endocytosis-related membranous proteins and exosomal markers (Tsg-101, CD9, and CD63) were enriched in the exosomes of both HS and control groups (Fig. 1C). Overall, the results indicated that these isolated vesicles had features mostly consistent with those of exosomes.

### Altered HS exosomal protein profiles revealed by iTRAQ-based proteomic analysis

**Figure 2:**
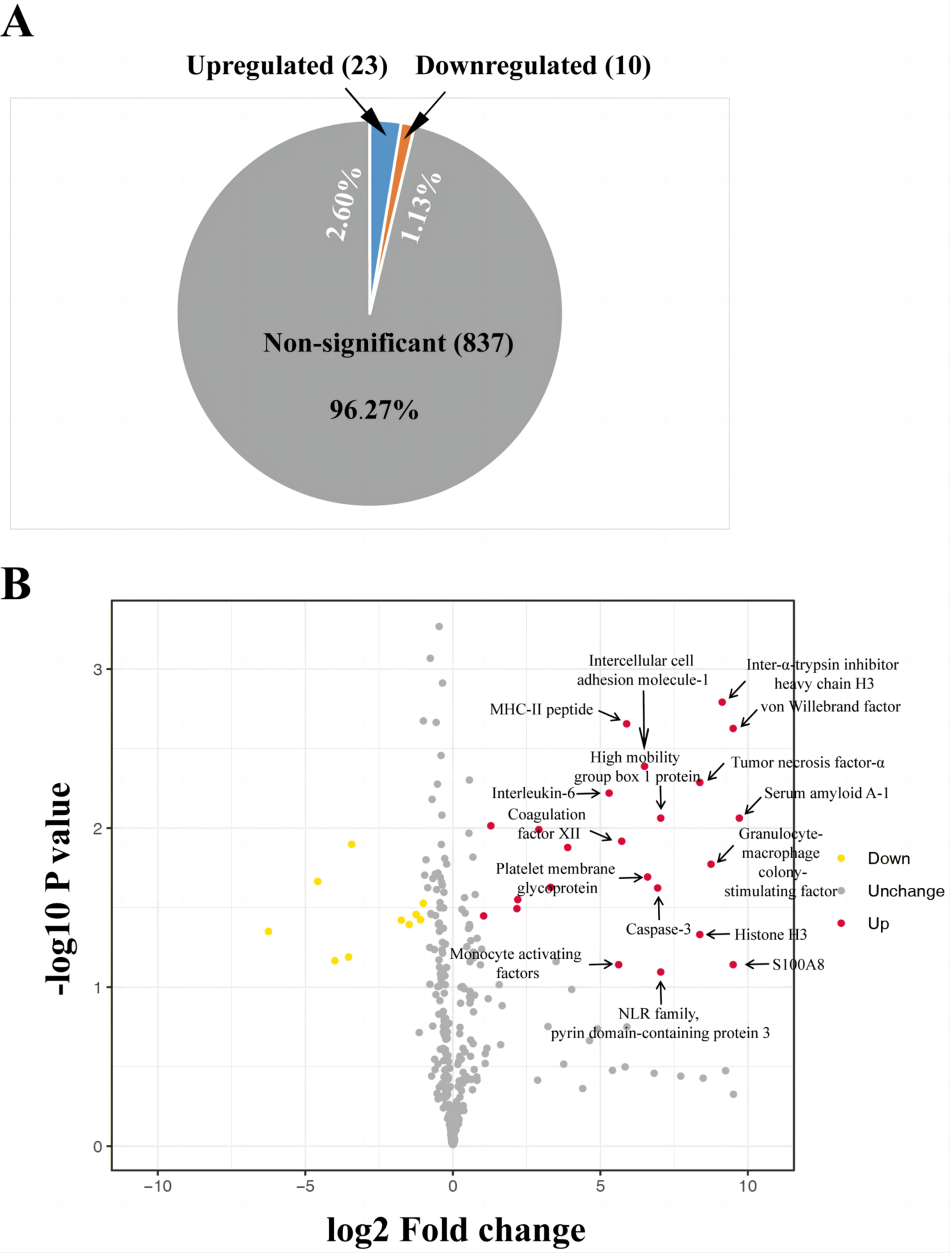
Heat stroke (HS) altered the synthesis patterns of proteins in plasma exosomes based on the analysis using iTRAQ. (A) The proportion and quantity of differentially regulated exosome proteins (non-significant, upregulated, and downregulated) isolated from the HS group in comparison with the control group (*n* = 6 in the control group and *n* = 10 in the HS group). Numbers denote proportions. Database: HomoSapiens. (B) Volcano plot to represent the change in protein folding in the plasma exosomes of the control and HS groups. X-axis presents the fold changes of proteins (HS/Ctrl; Log_2_) and the Y-axis gives −log_10_ *P* values. *P* values were measured by performing Student’s *t*-test (red: upregulated, gray: unchanged, and green: downregulated).

The iTRAQ-based proteomic analysis of the serum exosomes from the control and HS groups identified a total of 7,174 peptides corresponding to 884 proteins (Table S1), most of which had comparable enrichment (96.27%; Fig. 2). Nonetheless, the HS group showed upregulation of 23 exosomal proteins (2.60%) and downregulation of 10 proteins (1.13%) compared with the control group (Fig. 2A, Table S1). A volcano map was plotted to illustrate the fold-change (FC) of exosomal protein levels in the HS group compared with the control group (<0.6 or >1.5) (Fig. 2B).

**Figure 3:**
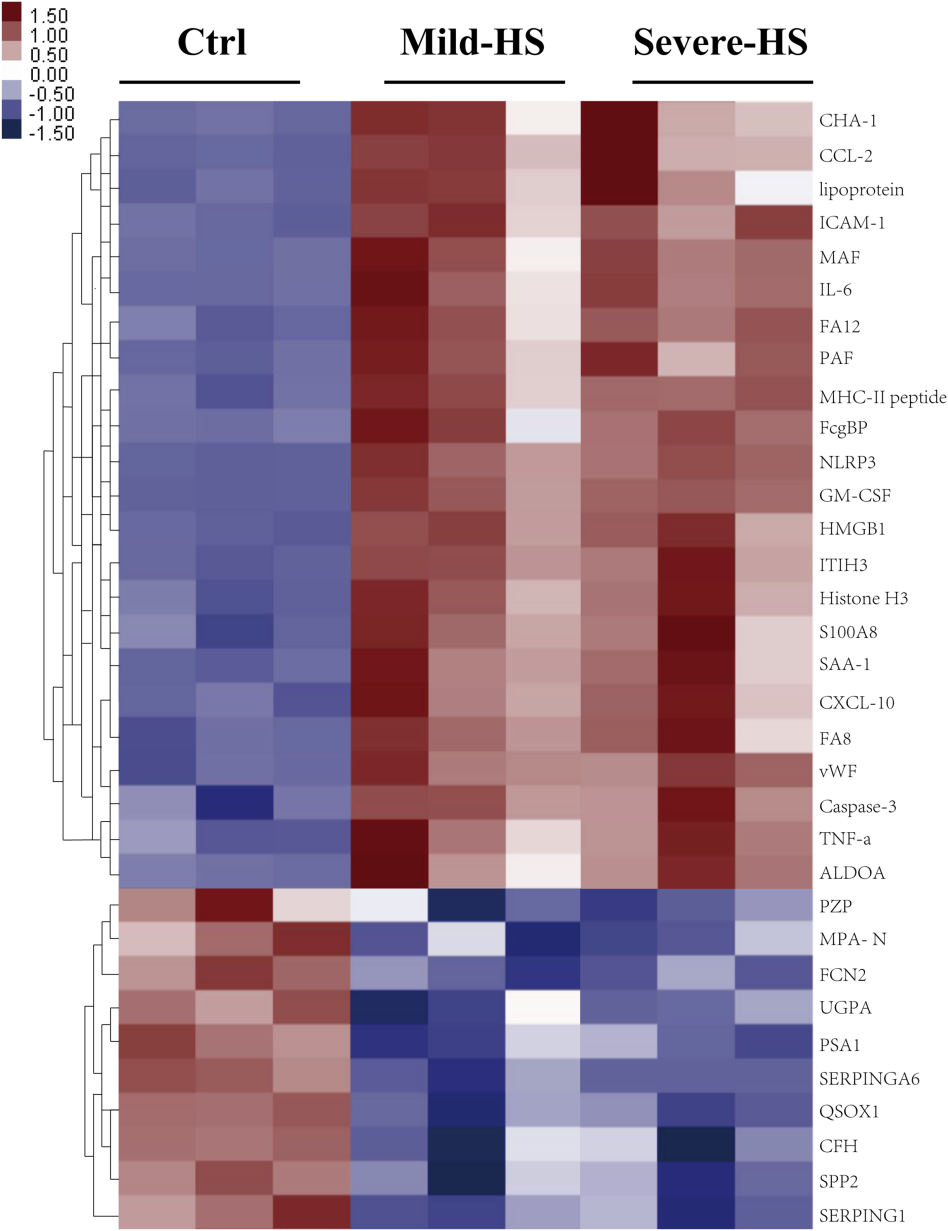
Cluster analysis of the elevated protein components from plasma exosomes in HS. The heat map shows the list of the 33 highly upregulated proteins (Ctrl/HS group). Various proteins showed high expression in the plasma exosomes from patients with HS. Database: go_201504.obo; website: www.geneontology.org, https://www.kegg.jp/.

A cluster analysis was conducted on differentially expressed proteins according to stimulants and samples for testing the creditability of the selected target protein. The levels of the 23 remarkably upregulated proteins were different between the control and HS groups with a low SD among the three sample sets (Fig. 3), indicating the high repeatability of results. Several remarkably upregulated exosomal proteins were identified in the HS group (Table 1), including SAA-1, vWF, tumor necrosis factor (TNF)-α, granulocyte–macrophage colony-stimulating factor, inter-α-trypsin inhibitor heavy chain H3, S100A8, histone H3, caspase-3, pyrin domain-containing protein 3, high mobility group box 1 protein, NLR family, intercellular cell adhesion molecule-1, platelet membrane glycoprotein, MHC-II peptide, MFG-E8, interleukin (IL)-6, monocyte activating factors, chemokine (C-C motif) ligand-2, chemokine (C-X-C motif) ligand-10, Fc-fragment of IgG binding, and immunoglobulin heavy constant Δ. These were closely related to acute phase response, platelet degranulation and activation, inflammation/immune regulation, cell injury, death, and adhesion. Furthermore, exosomal SAA-1, vWF, platelet membrane glycoprotein, S100A8, and histone H3 were more enriched in patients with severe HS than in those with mild HS (Table 1).

**Table 1:** Upregulated plasma exosomal proteins in HS and the main related pathways.

Differentially regulated proteins (HS *vs*. control)	Fold change	FDR (%)	Uniprot number	Main related pathways	Differentially regulated proteins (severe-HS *vs*. mild-HS)	Fold change
Serum amyloid A-1	59.69	0.59	P0DJI8	Immunity/inflammation response	Serum amyloid A-1	14.85
von Willebrand factor	57.22	0.55	P04275	Platelet activation	von Willebrand factor	9.96
S100A8	42.53	0.55	P05109	Immunity/inflammation response	S100A8	7.54
Inter-α-trypsin inhibitor heavy chain H3	29.95	0.58	Q06033	Acute phase response	Histone H3	4.59
Granulocyte-macrophage colony-stimulating factor	24.78	0.77	P04141	Immunity/inflammation response	Platelet membrane glycoprotein	4.50
Tumor necrosis factor-α	21.14	0.77	P01375	Immunity/inflammation response		
Histone H3	16.32	0.77	P0DPK2	Immunity/inflammation response		
High mobility group box 1 protein	13.52	0.78	P09429	Immunity/inflammation response		
Caspase-3	11.31	0.77	P42574	Programmed cell death		
NLR family, pyrin domain-containing protein 3	9.34	0.77	P0DMW2	Immunity/inflammation response		
Intercellular cell adhesion molecule-1	7.32	0.81	P05362	Cell adhesion		
Platelet membrane glycoprotein	3.18	0.63	P40197	Platelet activation		
MHC-II peptide	3.07	0.63	P01903	Immunity/inflammation response		
Coagulation factor XII	2.97	0.77	P00748	Platelet activation		
Monocyte activating factors	2.29	0.71	P13500	Immunity/inflammation response		
Interleukin-6	2.23	0.77	P08505	Immunity/inflammation response		
Chemokine (C-C motif) ligand 2	2.14	0.77	F1N4S8	Immunity/inflammation response		
Coagulation factor VIII	2.14	0.77	P00451	Platelet activation		
Chemokine (C-X-C motif) ligand -10	1.97	0.71	O55038	Immunity/inflammation response		
Immunoglobulin heavy constant Δ	1.91	0.77	P01880	Immunity/inflammation response		
Fructose-bisphosphate aldolase A	1.77	0.77	P04075	Glycolysis		
Carbonic anhydrase 1	1.76	0.75	P00915	Acute phase response		
Lipoprotein	1.76	0.77	P06858	Lipid metabolism		

**Note:**

HS, heat stroke; FDR, false discovery rate.

**Figure 4:**
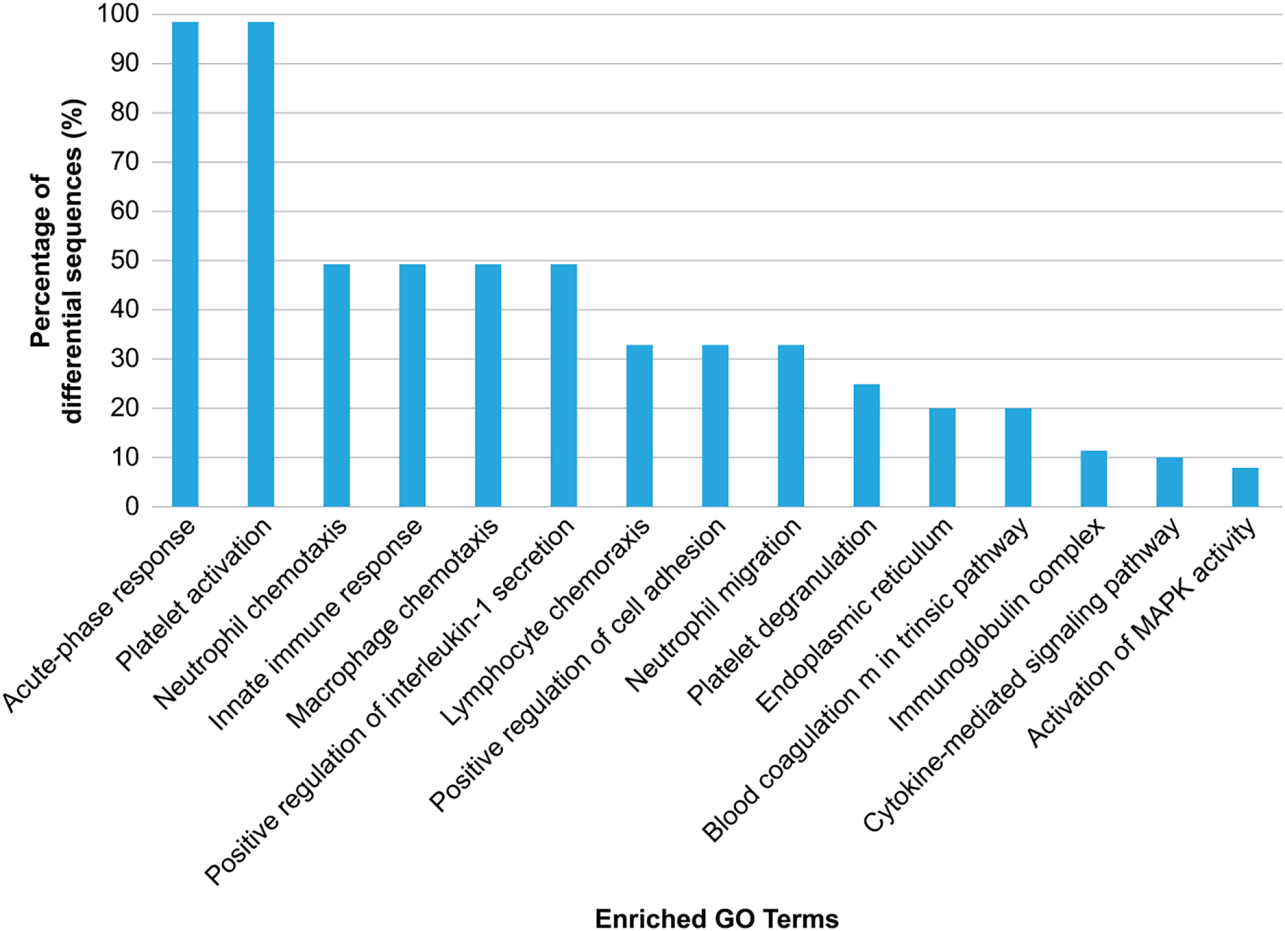
Gene Ontology (GO) enrichment analysis of the differentially regulated exosome proteins. The top 15 enriched terms in accordance with the GO functional annotation clustering of the 33 differentially regulated proteins in the plasma exosomes from patients with heat stroke and the proportions of the involved sequences are presented.

GO analysis was conducted to explore the biological effects of iTRAQ-identified exosomal proteins. The analysis revealed the 15 top terms connected with the downregulated and upregulated exosomal proteins in the case of HS (Fig. 4), and different protein enrichment significances for all clusters. Clusters with the highest upregulated levels were associated with various terms, including acute phase response, platelet activation/degranulation, innate immune response, neutrophil/macrophage/lymphocyte chemotaxis, neutrophil migration, positive IL-1 production regulation, positive cell adhesion regulation, and endoplasmic reticulum, and had a close relationship with the clinical characteristics of HS.

**Figure 5:**
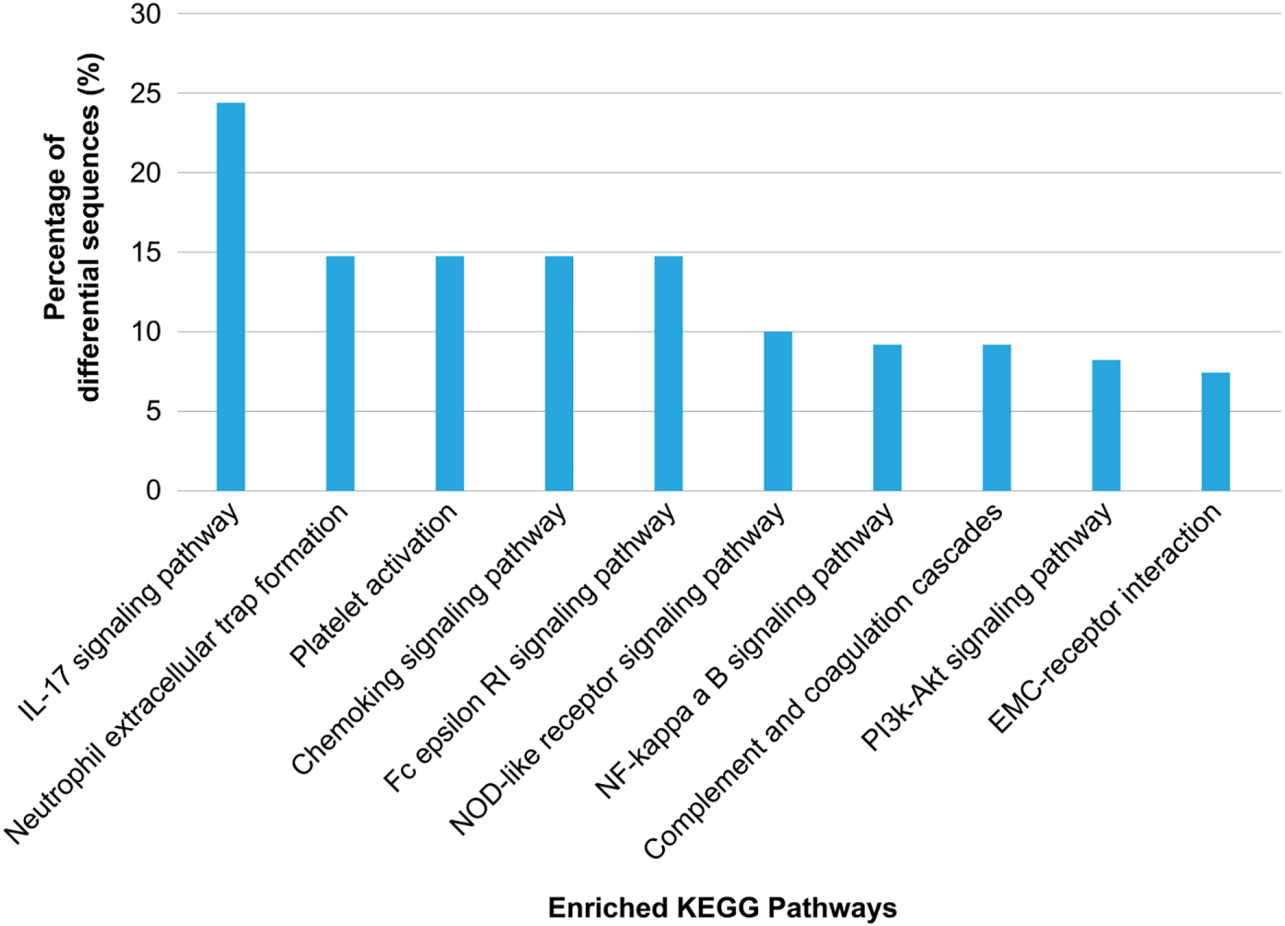
Kyoto Encyclopedia of Genes and Genomes (KEGG) pathway analysis of the differentially regulated exosome proteins. The 10 major KEGG pathways enriched in the plasma exosomes from patients with heat stroke and the proportions of the involved sequences in all pathways are given.

KEGG annotation showed that the enriched proteins were involved in pathways such as platelet activation, coagulation, and inflammation and immunity response, including platelet activation, IL-17 pathway, chemokine pathway, Fc epsilon RI pathway, NF-κ B pathway, neutrophil extracellular trap formation, NOD-like receptor pathway, ECM-receptor interaction, phosphatidylinositol 3′-kinase-Akt pathway, and complement and coagulation cascades (Fig. 5). Serum exosomes were associated with HS-mediated damage by activating relevant pathways in target cells. The most remarkably upregulated exosomal proteins in HS related to immunity and inflammation response and platelet activation-related pathways are listed in Table 1.

### Basic feature comparisons between HS and control groups

The patients with HS and participants in the control group comprised of males with very limited concurrent diseases and without any previous use of medication. The average ages of subjects in all three groups were similar: 26.90 ± 3.80 years old in the control group, 27.93 ± 3.86 years old in the mild HS group, and 27.43 ± 3.91 years old in the severe HS group. The total 28-day mortality was 16.0% (8/50) among HS patients; it was much higher in the group with severe HS (38.1%) than in the group with mild HS (0%, *P* < 0.001). ICU stay was significantly longer for patients with severe HS than for those with mild HS (median: 4 *vs*. 14 days, *P* < 0.001). The core body temperatures upon admission were comparable between patients with mild HS (38.26 ± 1.30 °C) and those with severe HS (38.98 ± 1.89 °C). Furthermore, both groups showed significantly higher temperatures than the control group (36.57 ± 0.47 °C, both *P* < 0.001). In addition, the overall disease severity and organ dysfunction, evaluated using SOFA (11.29 ± 5.71 *vs*. 3.76 ± 2.36, *P* < 0.001) and APACHE II (16.86 ± 4.22 *vs*. 4.69 ± 4.03, *P* < 0.001) scores, were higher in the severe HS group than that in the mild HS group (Table 2). None of the participants had taken prophylactic heparin, antibiotics, or fresh frozen plasma/red blood cells/platelet transfusion upon admission prior to sample extraction.

**Table 2:** Basic features and disease severity scores of study subjects.

Features	Control subjects (*N* = 30)	Patients with mild HS (*N* = 29)	Patients with severe HS (*N* = 21)	*P*-value
Age (y)	26.90 ± 3.80	27.93 ± 3.86	27.43 ± 3.91	0.5914
ICU mortality (%)	0 (0/30)	0 (0/29)	38.1 (8/21)^†††^^,^ ^§§§^	<0.001
Length of ICU stay (days), median (interquartile range)	0 (0)	4 (3–6)^†††^	14 (10–18.75)^†††^^,^ ^§§§^	<0.001
T (°C)	36.57 ± 0.47	38.26 ± 1.30^†††^	38.98 ± 1.89^†††^	<0.001
APACHE II score	0.47 ± 0.82	4.69 ± 4.03^†††^	16.86 ± 4.22^†††^^,^ ^§§§^	<0.001
SOFA score	0.60 ± 0.62	3.76 ± 2.36^†††^	11.29 ± 5.71^†††^^,^ ^§§§^	<0.001

**Notes:**

HS, heat stroke; ICU, intensive care unit; T, temperature; APACHE, acute physiology and chronic health evaluation; SOFA, sequential organ failure assessment.

††† *P* < 0.001 *vs*. normal controls.

§§§ *P* < 0.001 *vs*. mild-HS group.

According to the routine biochemical tests carried out upon admission, there were significant differences observed in organ dysfunction-related biochemical indicators between patients with mild and severe HS. These included respiratory impairment, hemodynamic instability, decreased kidney function, abnormal coagulation functions, rhabdomyolysis, liver injury indices, cardiac injury index, CNS disorders, and elevated inflammatory marker procalcitonin. Moreover, the differences in heart rate, mean arterial pressure (MAP), levels of blood urea nitrogen (BUN), fibrin (Fib), and white blood cell (WBC) counts were not significant between the mild and severe HS groups (Table 3). Most organ dysfunction parameters (except for MAP, BUN, and WBC) were significantly higher in the severe HS group than in the healthy control group. PaO_2_/FiO_2,_ PCT, Fib, PLT, MYO, and GCS were significantly higher in the mild HS group than in the healthy control group.

**Table 3:** Laboratory and clinical results between HS cases and control subjects.

Features	Control subjects (*N* = 30)	Subjects with mild HS (*N* = 29)	Subjects with severe HS (*N* = 21)	*P*-value
Hemodynamic data				
HR (beats/min)	75.70 ± 7.89	82.62 ± 22.54	96.86 ± 31.61^††^	0.0039
MAP (mmHg)	80.10 ± 7.32	78.59 ± 9.51	71.62 ± 20.56	0.0583
Vasoactive drug, *n* (%)	0 (0)	0 (0)	10 (37.5)^†††^^,^ ^§§§^	<0.001
Lactate (μmol/L)	1.02 ± 0.46	1.58 ± 0.92	3.59 ± 3.32^††††^^,^ ^§§§^	<0.001
Ventilatory data				
PaO_2_/FiO_2_	341.7 ± 35.82	296.3 ± 57.82^††^	279.7 ± 60.80^††††^	<0.001
MV, n (%)	0 (0)	0 (0)	12 (57.1)^†††^^,^ ^§§^	<0.001
Inflammatory data				
WBC (×10^9^ cells/L)	9.44 ± 3.08	11.45 ± 6.05	10.98 ± 4.24	0.2371
PCT (ng/ml)	0.33 ± 0.33	2.10 ± 2.23^†^	4.03 ± 4.04^††††^^,^ ^§^	0.0211
IL-6 (pg/ml)	1.56 ± 0.70	111.9 ± 63.30	525.1 ± 448.6^††††^^,^ ^§§§§^	<0.001
Hepatic data				
ALT (U/L)	30.51 ± 14.39	52.69 ± 47.20	82.69 ± 47.20^††††^^,^ ^§^	<0.001
AST (U/L)	26.73 ± 12.35	112.3 ± 67.42	2,045 ± 3,791^††^^,^ ^§§^	<0.001
TBil (µmol/L)	17.57 ± 14.44	62.41 ± 91.32	92.90 ± 26.93^††^^,^ ^§§^	0.001
ALB (g/L)	41.29 ± 5.29	40.06 ± 4.21	37.30 ± 3.63^††^	0.0046
Renal data				
Cr (μmol/L)	92.9 ± 26.93	106.7 ± 31.62	164.4 ± 83.51^††††^^,^ ^§§§^	<0.001
BUN (mmol/L)	6.17 ± 2.21	6.06 ± 2.22	8.23 ± 6.62	0.0899
Urine output (ml/d)	2,630 ± 7,16.4	2,878 ± 918.0	2,008 ± 1,394^§§^	0.116
CRRT, *n* (%)	0 (0)	0 (0)	12 (57.1)^††^^,^ ^§§^	<0.001
Coagulation data				
PT (s)	13.99 ± 1.05	16.91 ± 3.15	25.50 ± 15.23^††††^^,^ ^§§^	<0.001
INR	1.27 ± 0.27	1.40 ± 0.32	2.59 ± 2.20^†††^^,^ ^§§^	<0.001
Fib (g/L)	3.78 ± 0.70	2.42 ± 0.56^††††^	2.01 ± 0.76^††††^	<0.001
PLT (×10^9^/L)	227.1 ± 65.74	172.3 ± 67.37^††^	99.95 ± 60.06^††††^^,^ ^§§§^	<0.001
D-dimer	1.08 ± 1.03	2.10 ± 2.26	15.76 ± 5.80^††††^^,^ ^§§§§^	<0.001
FDP	6.88 ± 2.83	8.90 ± 19.65	54.97 ± 54.05^††††^^,^ ^§§§§^	<0.001
Rhabdomyo data				
CK (μg/L)	59.33 ± 23.66	955.2 ± 1320	4,920 ± 5,839^††††^^,^ ^§§§§^	<0.001
MYO (μg/L)	46.48 ± 13.45	503.9 ± 713.9 ^†^	1,330 ± 961.2^††††^^,^ ^§§§§^	<0.001
Cardiac data				
CK-MB	3.76 ± 2.07	5.96 ± 4.33	39.05 ± 49.36^††††^^,^ ^§§§§^	<0.001
cTnI	14.26 ± 9.88	71.70 ± 64.61	834.6 ± 1,120^††††^^,^ ^§§§§^	<0.001
CNS data				
GCS score	15 ± 0	13.14 ± 2.88^†^	8.24 ± 4.18^††††^^,^ ^§§§§^	<0.001

**Notes:** ALB, albumin; Cr, creatinine; CK, creatine kinase; BUN, blood urea nitrogen; AST, aspartate aminotransferase; ALT, alanine aminotransferase; CNS, central nervous system; CK-MB, CK-myocardial band; FDP, fibrin degradation product; cTnI, cardiac troponin I; HR, heart rate; Fib, fibrin; GCS, Glasgow Coma Scale; FiO_2_, percentage of inspired oxygen; MAP, mean arterial pressure; INR, international normalized ratio; MYO, myoglobin; MV, mechanical ventilation; PCT, procalcitonin; IL, interleukin; PT, prothrombin time; PaO_2_, partial pressure of arterial oxygen; TBil, total bilirubin; PLT, platelet; WBC, white blood cell.

† *P* < 0.05.

†† *P* < 0.01.

††† *P* < 0.001.

†††† *P* < 0.0001 *vs*. control subjects.

§ *P* < 0.05.

§§ *P* < 0.01.

§§§ *P* < 0.001.

§§§§ *P* < 0.0001 *vs*. subjects with mild heat stroke.

### Enrichment of plasma exosomal histone vWF, SAA-1, and S100A8 in HS patients

**Figure 6:**
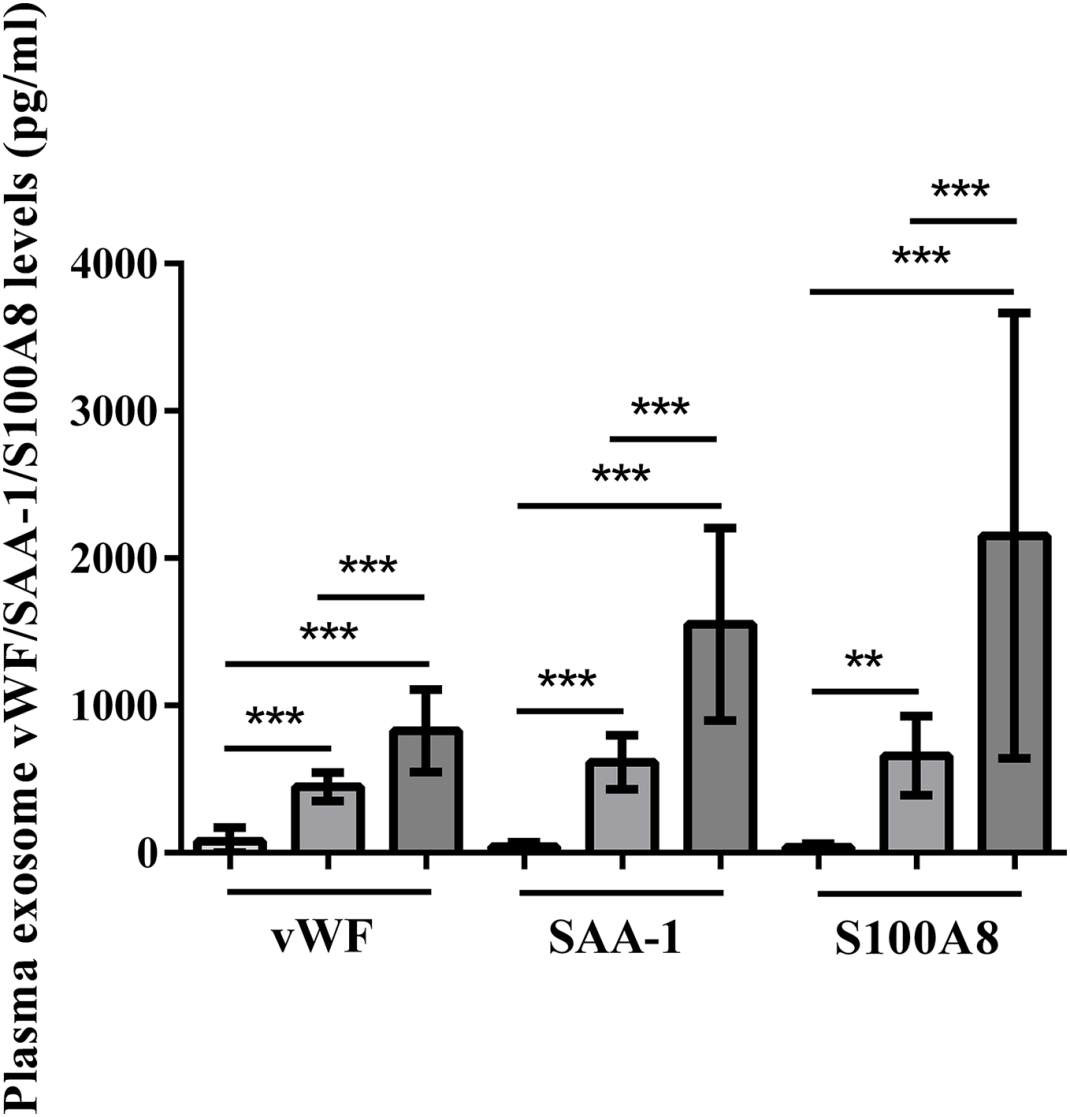
vWF, SAA-1, and S100A8 levels in plasma exosomes of healthy controls and patients with mild or severe heat stroke (HS) upon admission. Plasma exosomal levels of vWF, SAA-1, and S100A8 were greatly higher in the group with severe HS than in the group with mild HS and the healthy controls. Plasma exosomal levels of vWF, SAA-1, and S100A8 in the group with severe HS were considerably higher than those in the group with mild HS. **P < 0.01, ***P < 0.001 were used in the *post-hoc* test and one-way ANOVA. All data are given in the form of mean ± SEM.

Plasma exosomal vWF, SAA-1, and S100A8 levels at admission were significantly higher in HS patients than in control subjects (*P* < 0.001). Furthermore, the severe HS group had higher levels of these proteins than the mild HS group (*P* < 0.001) (Fig. 6).

### Correlations of plasma exosomal vWF, SAA-1, and S100A8 with disease severity and organ function in HS

Plasma exosomal vWF, SAA-1, and S100A8 levels at admission showed a remarkably positive correlation with the APACHE II score. vWF and SAA-1, rather than S100A8, were positively correlated with the SOFA score. There were also strong (vWF and SAA-1) and moderate (S100A8) positive correlations between the exosomal protein levels of these three proteins and indexes for assessing disseminated intravascular coagulation (ISTH) scores. The SAA-1 levels were positively correlated with the MELD score reflecting liver function (Table 4). Nonetheless, the associations of the above-mentioned exosomal proteins with individual organ function indicators were weak (r < 0.6).

**Table 4:** Correlation between plasma exosome vWF, SAA-1, and S100A8 levels and laboratory indicators.

	vWF		SAA-1		S100A8	
Laboratory indicators	r-value	*P*-value	r-value	*P*-value	r-value	*P*-value
APACHE II score	0.7300	<0.001	0.7743	<0.001	0.6514	<0.001
SOFA score	0.6905	<0.001	0.7809	<0.001	0.3789	<0.001
MAP (mmHg)	−0.1604	0.1553	−0.3859	<0.001	−0.0959	<0.001
Lactate (µmol/L)	0.5498	<0.001	0.5176	<0.001	0.1176	0.2989
PaO_2_/FiO_2_	−0.5045	<0.001	−0.5054	<0.001	−0.3546	0.0012
PCT (ng/ml)	0.4659	<0.001	0.4158	<0.001	0.6213	<0.001
MELD score	0.5656	<0.001	0.6847	<0.001	0.2373	0.0340
Cr (µmol/L)	0.4510	<0.001	0.6502	<0.001	0.1351	0.2321
ISTH score	0.8691	<0.001	0.8335	<0.001	0.6976	<0.001
CK (µg/L)	0.3907	<0.001	0.4807	<0.001	0.3379	0.0022
MYO (µg/L)	0.4831	<0.001	0.5393	<0.001	0.4486	<0.001
CK-MB	0.2759	0.0132	0.34	0.002	0.3183	0.0040
cTnI	0.4248	<0.001	0.4915	<0.001	0.4673	<0.001
GCS score	−0.5243	<0.001	−0.5693	<0.001	−0.5309	<0.001

**Note:**

APACHE, Acute Physiology and Chronic Health Evaluation; CK-MB, CK-myocardial band; CK, creatine kinase; Cr, creatinine; FiO2, percentage of inspired oxygen; cTnI, cardiac troponin I; MAP, mean arterial pressure; GCS, Glasgow Coma Scale; MYO, myoglobin; PaO2, partial pressure of arterial oxygen; PCT, procalcitonin; WBC, white blood cell; SOFA, Sequential Organ Failure Assessment.

**Figure 7:**
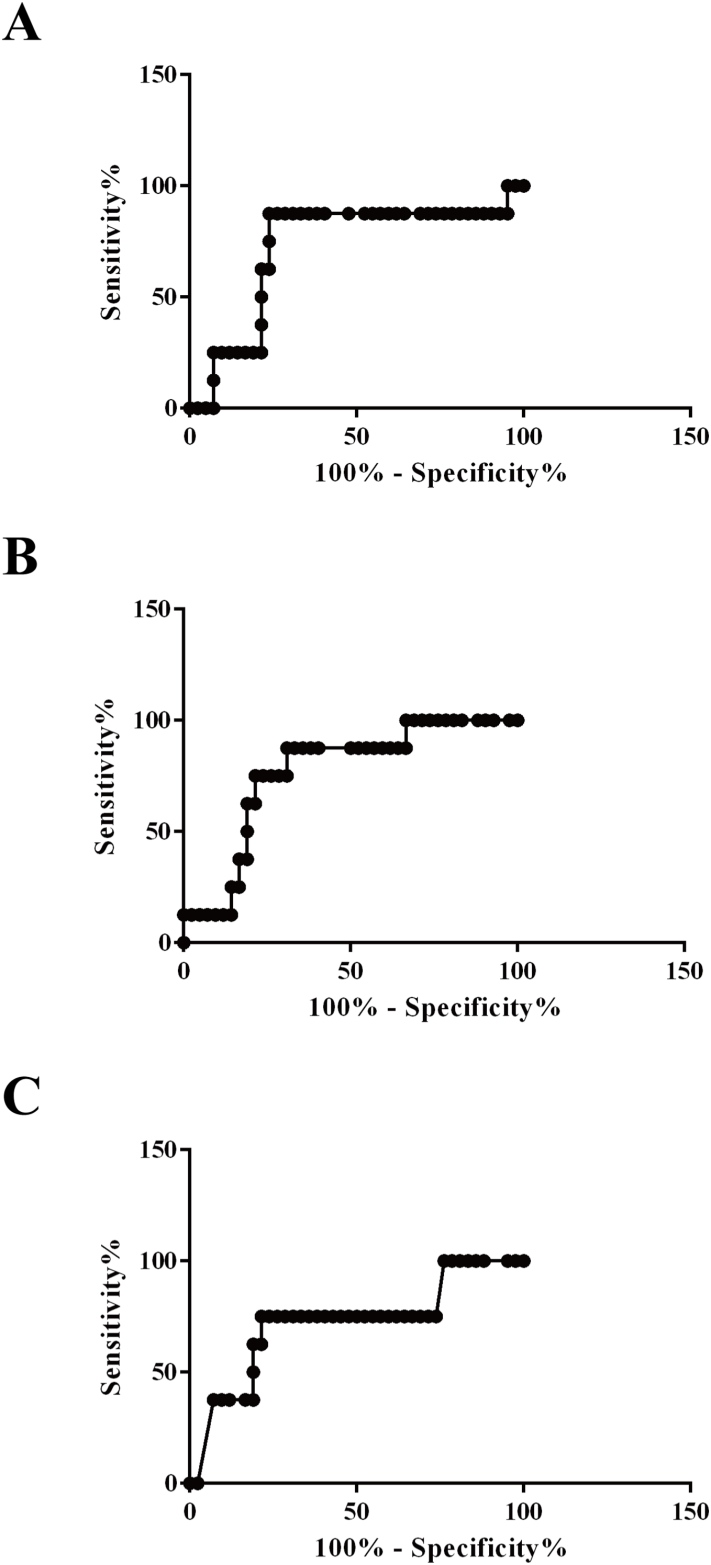
Applicability of using plasma exosomal levels of vWF, SAA-1, and S100A8 to distinguish non-survivors and survivors among HS patients. (A) Receiver operating characteristic (ROC) curve for plasma exosomal levels of vWF used to distinguish non-survivors and survivors among patients with HS (AU-ROC 0.7232; 95% confidence interval (CI) [0.5182–0.9282], *P* = 0.047). (B) ROC curve for plasma exosomal levels of SAA-1 used to distinguish non-survivors and survivors among patients with HS (AU-ROC 0.7649; 95% CI [0.6070–0.9227], *P* = 0.019). (C) ROC curve for plasma exosomal levels of S100A8 used to distinguish non-survivors and survivors among patients with HS (AU-ROC 0.7202; 95% CI [0.5110–0.9295], *P* = 0.050).

Area under the ROC curve values for plasma exosomal vWF, SAA-1, and S100A8 proteins to distinguish survivors from non-survivors were 0.7232 (95% confidence interval (CI) [0.5182–0.9282], *P* = 0.047), 0.7649 (95% CI [0.6070–0.9227], *P* = 0.019), and 0.7202 (95% CI [0.5110–0.9295], *P* = 0.050), respectively. Additionally, the sensitivity and specificity of the optimized cutoff value for predicting mortality risk for vWF (655 pg/100 μg), SAA-1 (1,184 pg/100 μg), and S100A8 (1,063 pg/100 μg) were 87.5%/76.19%, 75.0%/78.57%, and 75.0%/78.57%, respectively (Fig. 7).

## Discussion

This study conducted a complete evaluation of the expression profiles of plasma exosomal proteins to identify the differentially regulated proteins in patients with HS. Their corresponding enriched biochemical functions and molecular pathways were explored, which mainly revealed the acute phase response and activation of the immune/inflammatory system and platelets. Furthermore, it verified that several exosomal proteins showed undeniably higher levels in patients with HS, especially those with severe HS. These plasma exosomal proteins were tightly related to disease severity and organ dysfunction. Their levels cannot only help discriminate between severe and mild HS but also act as prognostic factors for death in the case of HS.

The mechanism of HS-related organ injury still needs to be elucidated. It is currently suggested that hyperthermal injury-mediated coagulation and inflammatory cascades have key effects compared with heat exposure-induced physical damage (Proctor et al., 2020; Liu et al., 2020). HS animal models show the pathological results of massive neutrophil infiltration, local microthrombosis in sinusoids, hepatocyte degeneration, ballooning, and cell membrane rupture around the liver sinusoids (Hifumi et al., 2018). At a core temperature of approximately 42.5–43 °C, HS baboon models display pathological results such as extensive leukocyte transmural migration, microvascular endothelial damage, excessive microthrombosis, interaction between endothelial leukocytes and platelets, and apoptosis in multiple organs (such as the gut, liver, spleen, lung, and kidney) (Bouchama et al., 2005; Roberts et al., 2008). Platelets actively respond to inflammatory stimulation and facilitate immune reaction by rendering a proinflammatory phenotype to the endothelium, increasing leukocyte accumulation and inflammation, and enhancing immune cell effector functions. In HS, the scheme of platelet activation and consequent thrombocytopenia has been reported (Geng et al., 2015). In this study, the profiling of plasma exosomal proteins revealed an enrichment of immune/inflammatory response and platelet activation pathways, suggesting their important role in the pathogenesis of HS. So far, studies regarding the exosomal proteome in HS are nearly absent. Our results were consistent with studies focusing on other critical diseases that are also characterized by an unbalanced inflammatory/immune response and subsequent multiple organ dysfunction. Proteome profiles of exosomes from septic patients presenting to the emergency department showed that, the expression of acute phase response proteins (especially serum amyloid A-1, C-reactive protein and serum amyloid A-2 proteins) and inflammatory response proteins (including immunoglobulin heavy constant ∆ and Fc-fragment of IgG binding protein) was up-regulated (Morris et al., 2020). It is worth mentioning that the incidence of coagulation abnormalities (with 34.8% of DIC) is reported to be much higher in HS than in sepsis. The enrichment of coagulation-, including platelet function pathway-, associated proteins in HS exosome prompts some possible unique pathological mechanisms, especially in the inflammation-coagulation crosstalk in HS.

Endothelial cell injury occurs in the early stage of HS and plays a key role in its pathogenesis (Yin et al., 2018). As a glycoprotein produced from stimulated platelets and injured endothelial cells, and as an important regulator for hemostasis, vWF can trap circulating platelets in the areas with vascular injury and regulate their subsequent accumulation and activation (Li et al., 2014). It not only participates in coagulation but also helps recruit leukocytes (Hifumi et al., 2018; Löf, Müller & Brehm, 2018) directly by binding to surface P-selectin glycoprotein ligand 1 to support their rolling and later, interaction with surface β2 integrin for promoting strong adhesion, or indirectly through the activated platelets. In presence of pathogens, vWF multimers can combine with neutrophil extracellular traps to form a network to aggregate leukocytes and platelets while promoting immunothrombosis (de Boer & Eikenboom, 2019). High vWF antigen levels have been reported to exist in trauma and are associated with increased endothelial permeability, persistently lower platelet counts, microthrombi formation, and organ failure (Yang et al., 2020). It has been proposed that vWF should be seen as a predictive factor for thrombotic risk and endothelial injury in COVID-19. Elevated vWF antigen levels can be detected in COVID-19 cases and are recommended to help estimate the death risk (Tiscia et al., 2020). As shown in a baboon model, HS increases the expression of vWF in various vital organs.

Acute phase response involves various physiological alterations during infection, inflammation, and trauma. The level of SAA, the most remarkably altered APR protein, may rise by 1,000-fold within 24 h (Sack, 2018). As discovered by Mithal et al. (2017) increased SAA levels in cord blood predict early neonatal sepsis in premature (29.7 weeks) babies. The role of SAA as a cytokine-like protein has been identified in intercellular communication; it provides feedback within immunological, inflammatory, protective, and neoplastic pathways. SAA can generate broad arrays of not only cytokines but also chemokines. Furlaneto & Campa (2000) employed recombinant human SAA to treat human blood neutrophils. According to the result, the treatment elevates the TNF-α, IL-8, and IL-1β levels by 75–400-fold in a lipid-independent manner. In 2007, El Kebir et al. (2007) discovered that SAA prolongs PMN cell lifespan and elongates PMN participation in inflammation.

As one of the S100 family members, S100A8 is a Ca^2+^-binding protein. S100A8 can be detected in monocytes and neutrophils as a Ca^2+^ sensor and is related to arachidonic acid metabolism and cytoskeleton rearrangement. In the case of inflammation, the increased release of S100A8 has important functions in regulating inflammatory reactions by promoting leukocyte recruitment and enhancing cytokine production. Therefore, S100A8 may be used as a diagnostic and therapeutic marker for inflammatory disorders. Blocking its activity with small-molecular antibodies or inhibitors in murine models can mitigate pathological conditions, implying the potential of the heterodimer as the therapeutic target (Wang et al., 2018).

This study demonstrated that exosomal vWF, SAA, and S100A8 were enriched in patients with HS, especially those with severe HS. Furthermore, as the levels of these proteins are positively related to disease severity and organ dysfunction, they can be used to differentiate between severe and mild HS, as well as help determine the prognosis of individual cases. As these three proteins show potent biological activities, they may have important effects on the pathogenesis of HS. Consequently, they are biomarkers for predicting disease prognosis and evaluating disease severity.

The current study has some limitations. This exploratory work had a small sample size, and the acquired findings were hypothesis-generating. Although it compared HS patients and healthy controls from multiple aspects, we only recruited HS patients with initial disease onset to prevent the influence of underlying diseases such as chronic inflammation and immunosuppression on the findings. This possibly limited the generalizability of the results. Another limitation was that only young males were included as severe heat stroke is more likely happen in highly intensified military training and physical exercise which man are more often involved. Furthermore, estrogen was proved to be protective in heat stroke induced injury. In addition, our hospital was a military hospital and nearly almost of the hospitalized severe heat stroke patients were male soldiers. The study was a singer center study, therefore, in the following research, we will consider to expand the participating centers and include female patients. Moreover, to compare the effects in non-infectious lesions *vs*. infectious lesions, we did not enroll any control subjects admitted to the ICU. Additionally, no functional or mechanistic protein studies was taken. We did not perform additional experiments to identify the specific roles of these differentially expressed proteins; thus, these markers need to be further explored in the future. Any of these proteins may be a potential target in treatment of HS-induced injury. Time-course and thoroughly step-by-step pathway study can be employed to clarify these issues. Finally, proteome profiling/relative mass spec-based quantitation usually shows larger run-to-run, sample-to-sample variations. In the following study, targeted mass spec-based methods (such as SRM or PRM) can be used to verify the candidate biomarkers.

## Conclusions

Plasma exosomes contain proteins associated with critical pathways related to a potential pathogenic mechanism of HS, such as acute phase response, platelet activation, and inflammatory response. Further functional studies are required to clarify the exact roles that such vesicles play in protein exchange and intercellular communication in HS.

## Supplemental Information

10.7717/peerj.16590/supp-1 Supplemental Information 1 Raw data of the transmission electron microscopy photo of exosomes in Figure 1A.

10.7717/peerj.16590/supp-2 Supplemental Information 2 Raw data of NTA analysis in Figure 1B.

10.7717/peerj.16590/supp-3 Supplemental Information 3 Raw data in Figure 1B.

10.7717/peerj.16590/supp-4 Supplemental Information 4 Raw data in Figure 1C.

10.7717/peerj.16590/supp-5 Supplemental Information 5 Raw data in Figure 6.

10.7717/peerj.16590/supp-6 Supplemental Information 6 Raw data in Figure 7.

10.7717/peerj.16590/supp-7 Supplemental Information 7 Raw data in Tables 2 and 3.

10.7717/peerj.16590/supp-8 Supplemental Information 8 Raw data in Table 4.

10.7717/peerj.16590/supp-9 Supplemental Information 9 The full list of all the identified 884 proteins.

10.7717/peerj.16590/supp-10 Supplemental Information 10 Venn diagram of heatstroke related exosomal proteins.

## List of Abbreviations

HS: Heat stroke
miRNAs: microRNAs
CNS: central nervous system
EDTA: ethylene diamine tetraacetic acid
PBS: phosphate-buffered saline
TEM: transmission electron microscopy
NTA: Nanoparticle tracking analysis
WB: Western blotting
iTRAQ: Isobaric tags for relative and absolute quantification
HPLC: High-performance liquid chromatography
HCD: higher-energy collisional dissociation
GO: Gene ontology
KEGG: Kyoto encyclopedia of genes and genomes
SD: standard deviation
IQR: interquartile ranges
ROC: Receiver operating characteristic
PAF: platelet-activating factor
WBC: white blood cell.

## Additional Information and Declarations

### DNA Deposition

The following information was supplied regarding the deposition of DNA sequences: The mass spectrometry proteomics data are available at the ProteomeXchange Consortium *via* the iProX partner repository: PXD040640.

### Data Availability

The following information was supplied regarding data availability: The raw data are available in the Supplemental Files.
