# Supplementary figures and images for: Proteomic profiling of serum exosomes reveals acute phase response and promotion of inflammatory and platelet activation pathways in patients with heat stroke
Source: PeerJ. 2023 Dec 13;11:e16590. doi: 10.7717/peerj.16590 (PMC10725172; doi:10.7717/peerj.16590)

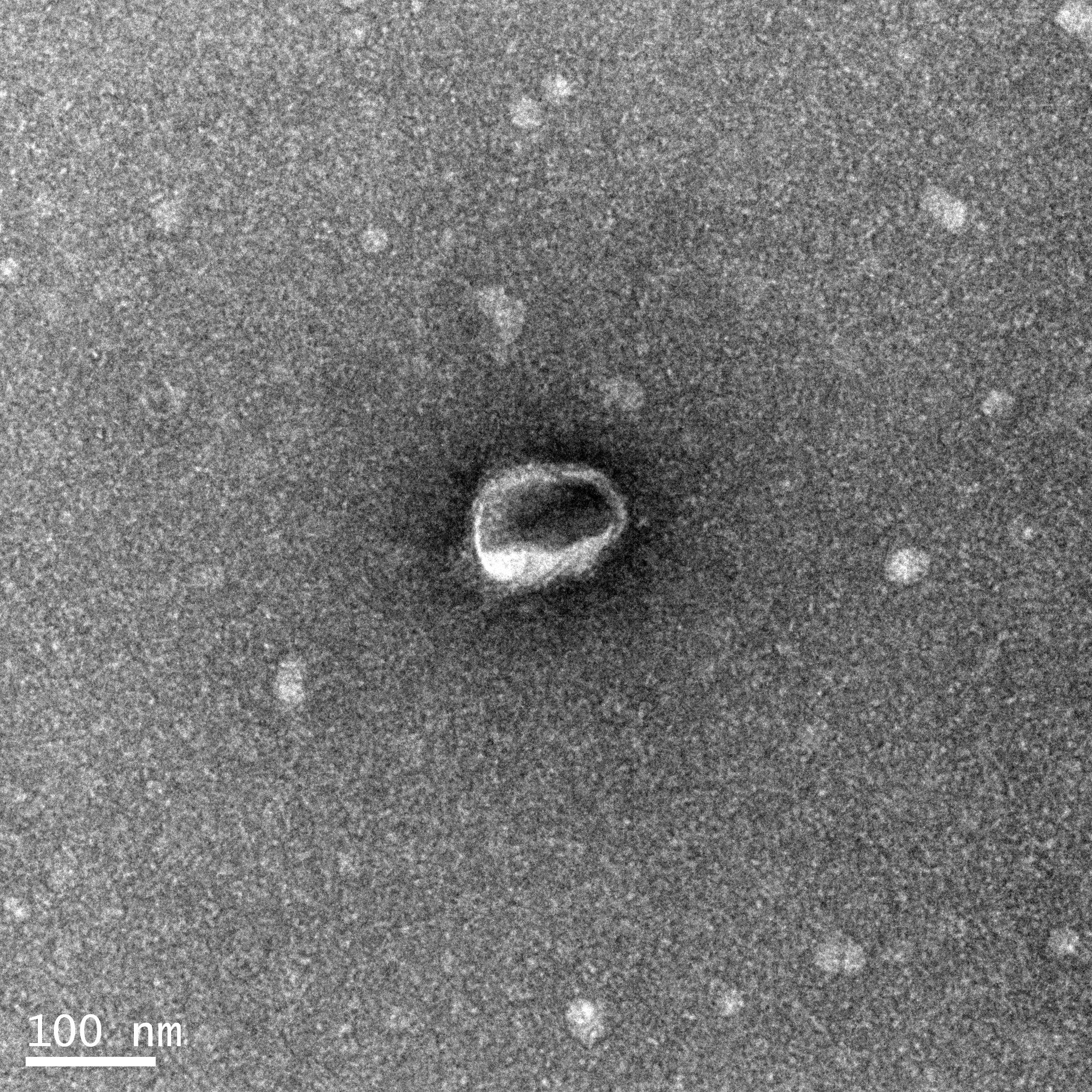

Supplement: Supplemental Information 1 [file peerj-11-16590-s001.zip › ╡τ╛╡/NC1-0004.jpg]

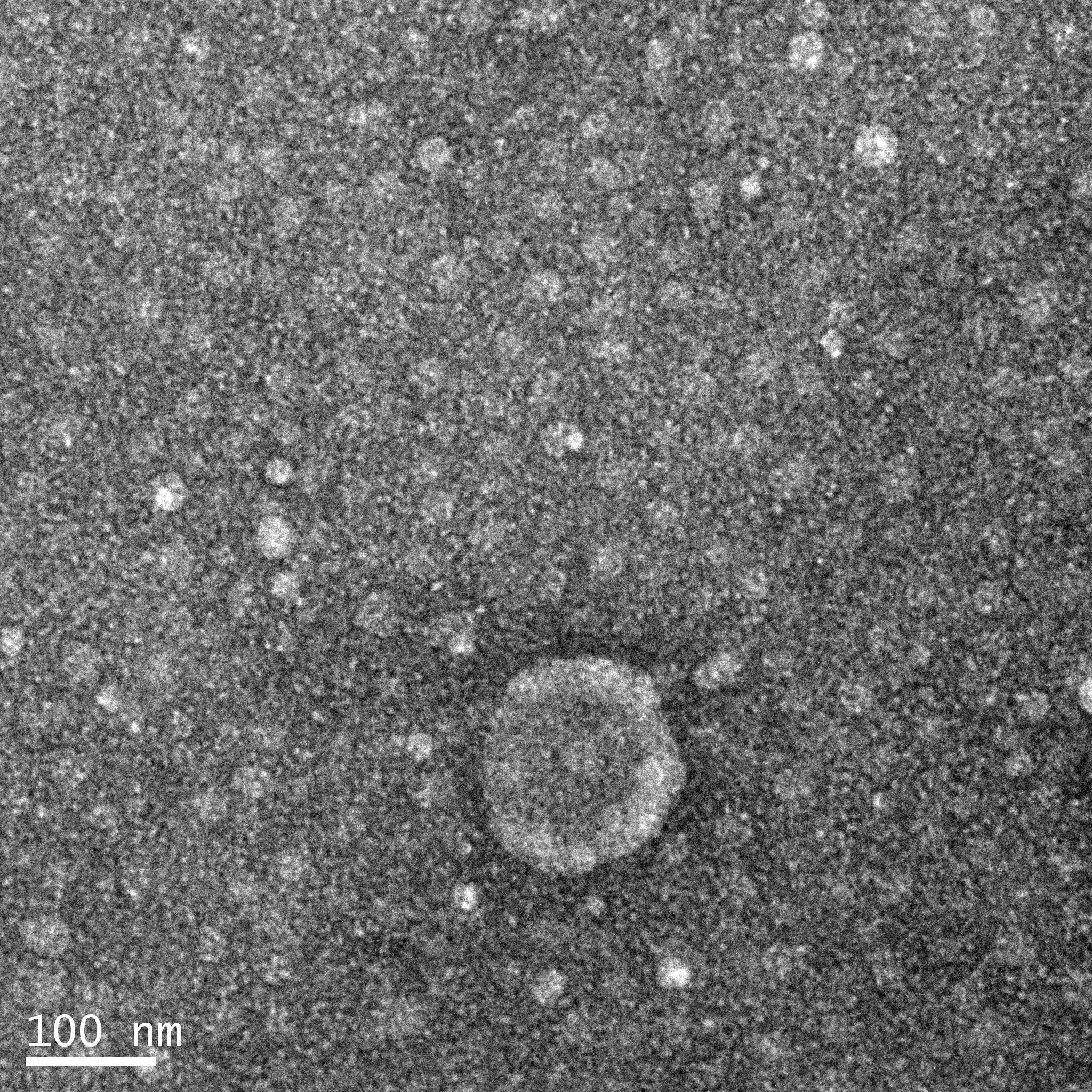

Supplement: Supplemental Information 1 [file peerj-11-16590-s001.zip › ╡τ╛╡/║═╝╤╨└-0002.jpg]

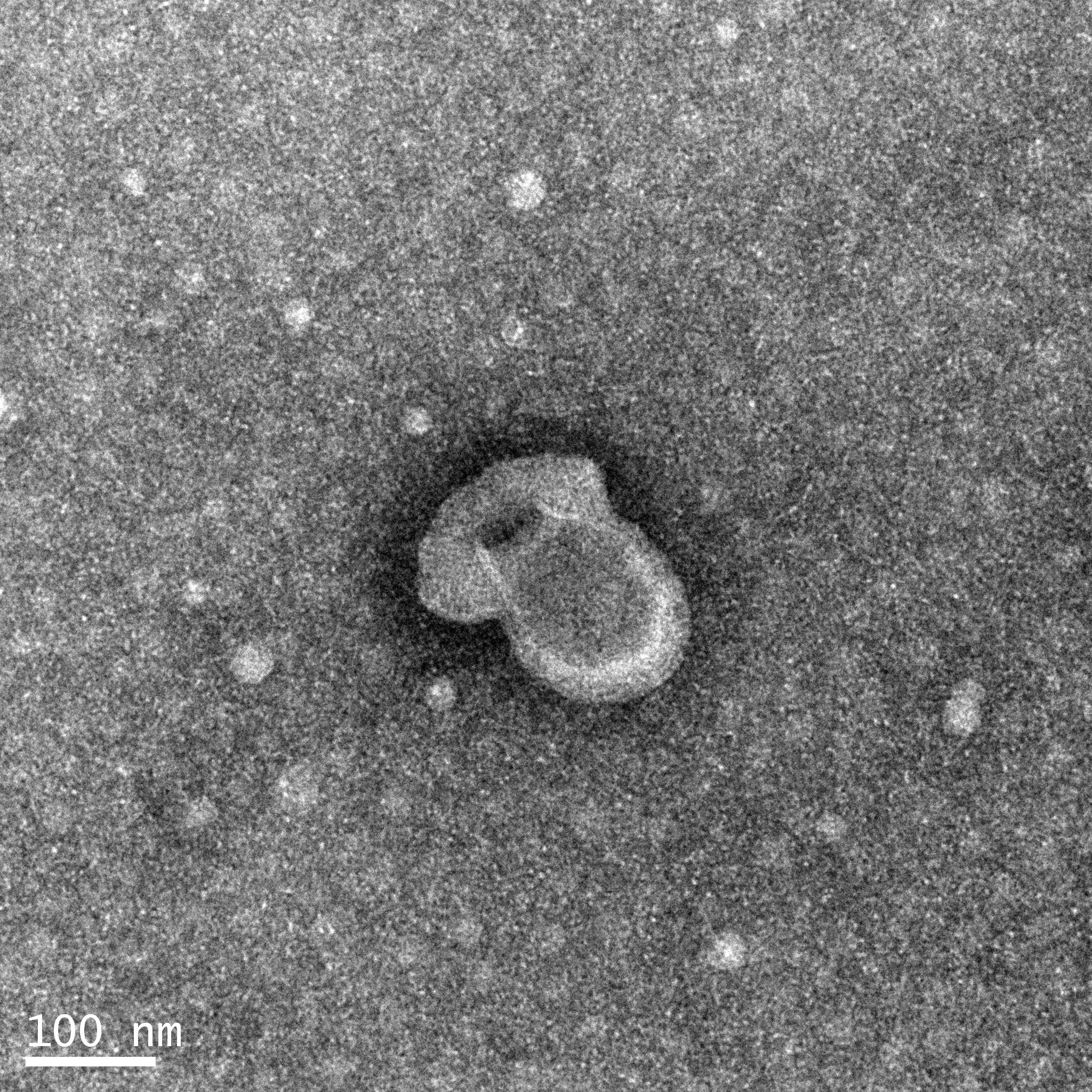

Supplement: Supplemental Information 1 [file peerj-11-16590-s001.zip › ╡τ╛╡/╤╧╒±╞μ-0002.jpg]

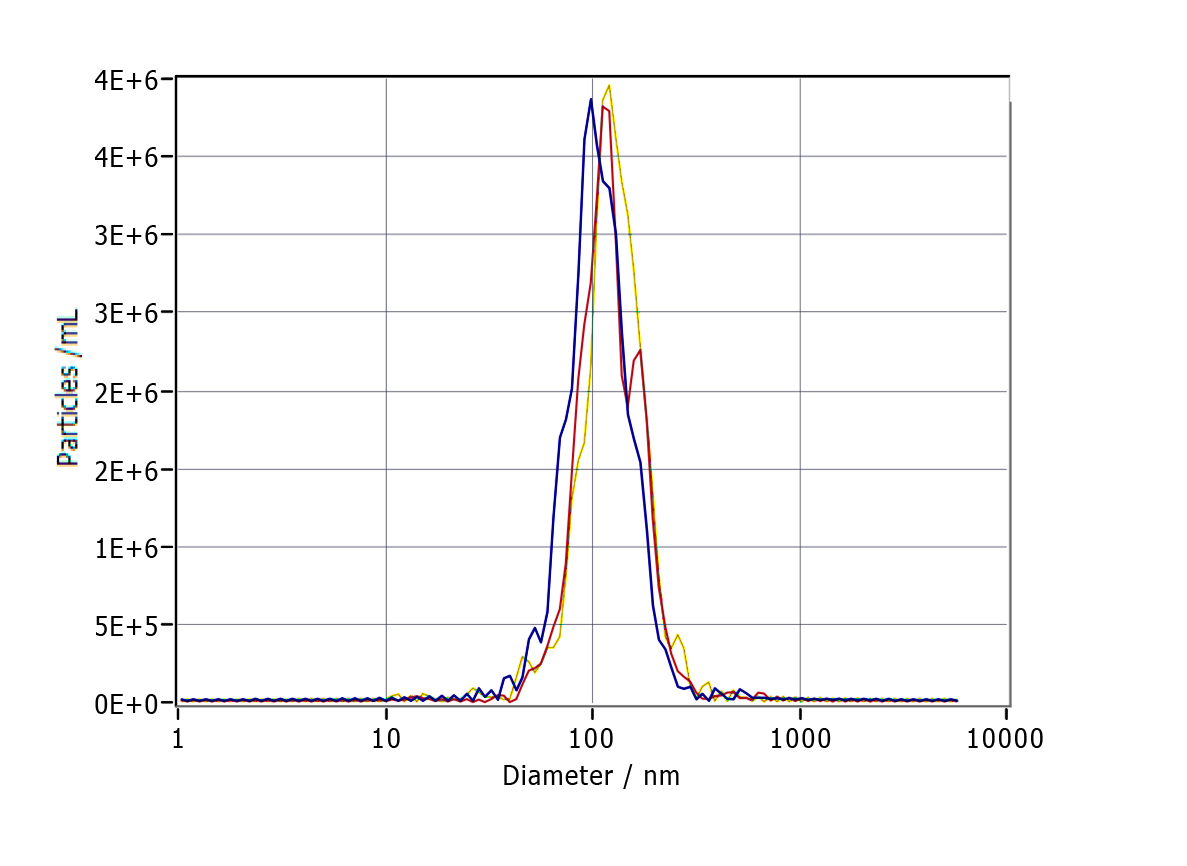

Supplement: Supplemental Information 2 [file peerj-11-16590-s002.jpg]

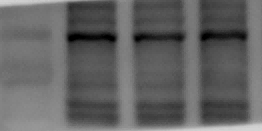

Supplement: Supplemental Information 4 [file peerj-11-16590-s004.zip › WPS═╝╞1⁄4┼·┴┐┤a└φ(1)/CD-63-1.png]

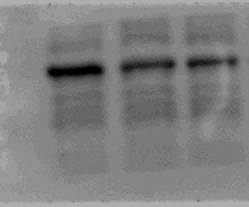

Supplement: Supplemental Information 4 [file peerj-11-16590-s004.zip › WPS═╝╞1⁄4┼·┴┐┤a└φ(1)/CD-63-2.png]

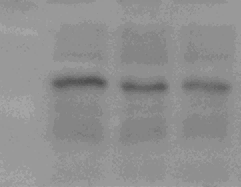

Supplement: Supplemental Information 4 [file peerj-11-16590-s004.zip › WPS═╝╞1⁄4┼·┴┐┤a└φ(1)/CD-63-3.png]

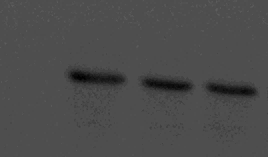

Supplement: Supplemental Information 4 [file peerj-11-16590-s004.zip › WPS═╝╞1⁄4┼·┴┐┤a└φ(1)/CD-9-1.png]

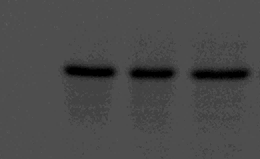

Supplement: Supplemental Information 4 [file peerj-11-16590-s004.zip › WPS═╝╞1⁄4┼·┴┐┤a└φ(1)/CD-9-2(1).png]

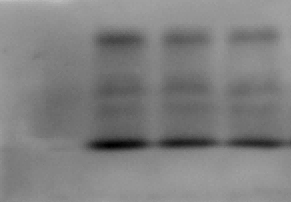

Supplement: Supplemental Information 4 [file peerj-11-16590-s004.zip › WPS═╝╞1⁄4┼·┴┐┤a└φ(1)/CD-9-3.png]

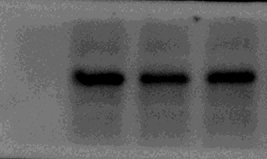

Supplement: Supplemental Information 4 [file peerj-11-16590-s004.zip › WPS═╝╞1⁄4┼·┴┐┤a└φ(1)/GADPH-1.png]

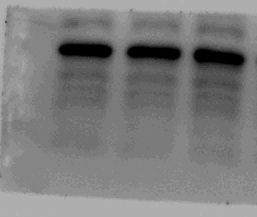

Supplement: Supplemental Information 4 [file peerj-11-16590-s004.zip › WPS═╝╞1⁄4┼·┴┐┤a└φ(1)/GADPH-2.png]

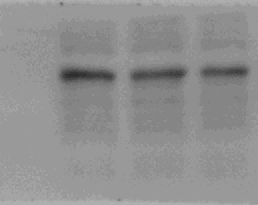

Supplement: Supplemental Information 4 [file peerj-11-16590-s004.zip › WPS═╝╞1⁄4┼·┴┐┤a└φ(1)/GADPH-3.png]

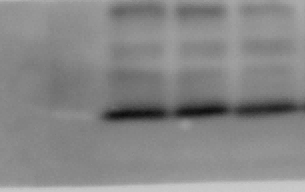

Supplement: Supplemental Information 4 [file peerj-11-16590-s004.zip › WPS═╝╞1⁄4┼·┴┐┤a└φ(1)/TSG101-1.png]

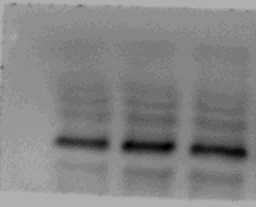

Supplement: Supplemental Information 4 [file peerj-11-16590-s004.zip › WPS═╝╞1⁄4┼·┴┐┤a└φ(1)/TSG101-2.png]

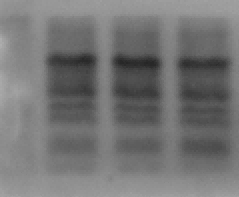

Supplement: Supplemental Information 4 [file peerj-11-16590-s004.zip › WPS═╝╞1⁄4┼·┴┐┤a└φ(1)/TSG101-3.png]

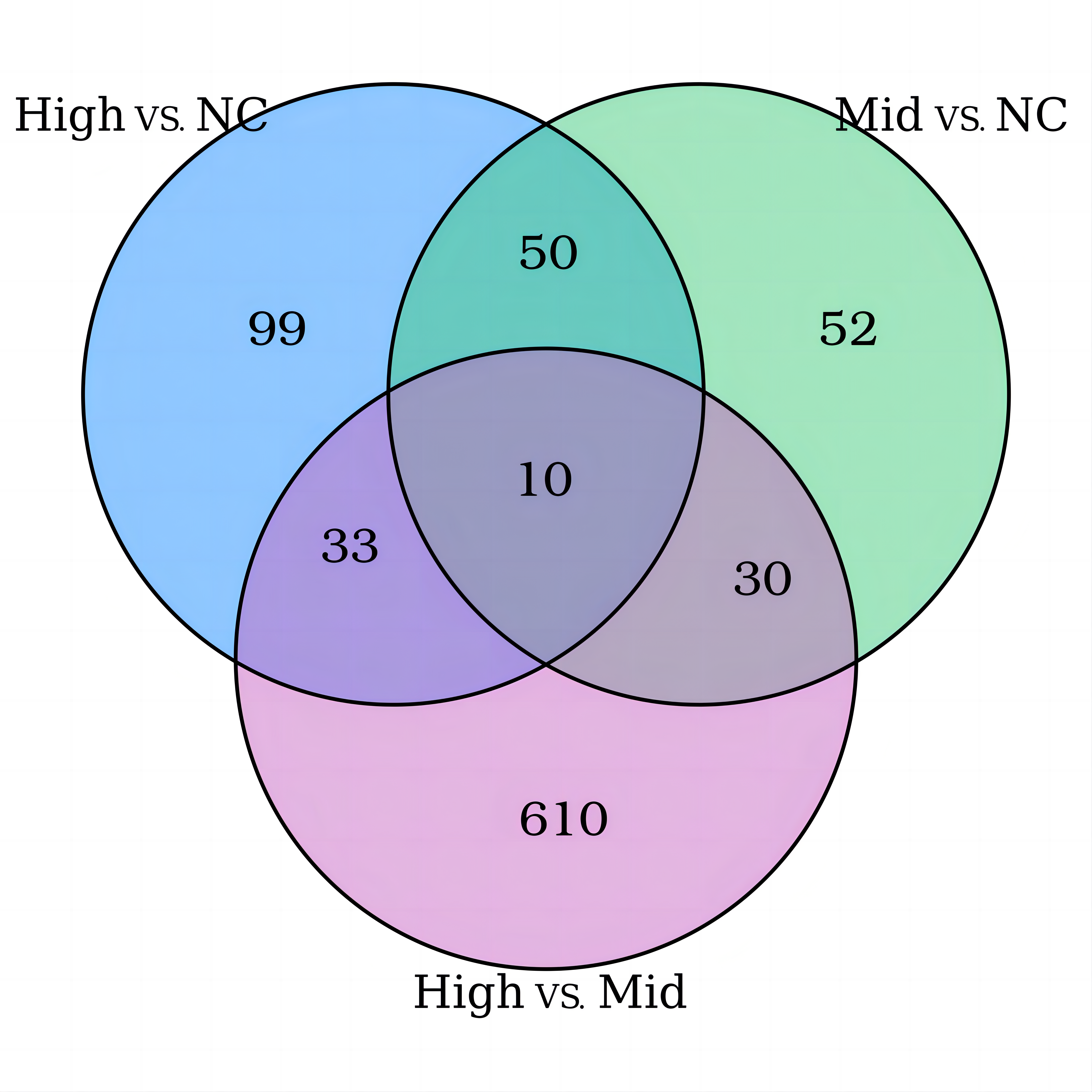

Supplement: Supplemental Information 10 [file peerj-11-16590-s010.png]
